# Effect of dose-delivery time for flattened and flattening filter-free photon beams based on microdosimetric kinetic model

**DOI:** 10.1371/journal.pone.0206673

**Published:** 2018-11-21

**Authors:** Hisashi Nakano, Daisuke Kawahara, Kaoru Ono, Yukio Akagi, Yutaka Hirokawa

**Affiliations:** 1 Hiroshima Heiwa Clinic, High-Precision Radiotherapy Center, Kawaramachi, Naka-ku, Hiroshima-shi, Hiroshima, Japan; 2 Devision of Radiation Oncology, Niigata University Medical and Dental Hospital, Asahimachi-dori, Chuo-ku, Niigata, Japan; 3 Radiation Therapy Section, Department of Clinical Support, Hiroshima University Hospital, Kasumi, Minami-ku, Hiroshima-shi, Hiroshima, Japan; 4 Medical and Dental Sciences Course, Graduate School of Biomedical and Health Sciences, Hiroshima University, Kasumi, Minami-ku, Hiroshima-shi, Hiroshima, Japan; North Shore Long Island Jewish Health System, UNITED STATES

## Abstract

The effect of dose-delivery time with flattening filter (FF) and flattening filter-free (FFF) photon beams based on microdosimetric kinetic model (MKM) was investigated in this study. Monte Carlo simulation with the particle and heavy ion transport code system (PHITS) was performed to calculate the dose-mean lineal energy y_D_ (keV/μm) of FF and FFF 6 MV photon beams using the IAEA phase-space files of Varian TrueBeam linear accelerator. Human non-small cell lung cancer NCI-H460 cells were used to determine the MKM parameters under the condition that dose-delivery times with continuous irradiation were 1, 5, 10, 30, and 60 min, and the adsorbed dose was 2, 4, and 8 Gy in this study. In addition, the relative biological effectiveness (RBE) of FF and FFF photon beams were calculated for evaluating the effect of dose delivery time. The RBE of FF decreased to 99.8% and 97.5% with 5 and 60 min for 2 Gy in comparison to 99.6% and 95.1% for 8 Gy, respectively. Meanwhile, that of FFF decreased to 99.5% and 94.9% with 5 and 60 min for 2 Gy in comparison to 99.5% and 94.9% for 8 Gy, respectively. Dose-delivery time has an effect on the RBE with photon beams. In other words, the dose-delivery time should be considered during radiation therapy. Furthermore, FFF photon beams were an effective irradiation method compared to FF in dose-delivery time on account of improving clinic throughput.

## Introduction

Flattening filter-free (FFF) photon beams provide an increased instantaneous dose of X-ray pulses compared with a conventional flattening filter (FF) photon beams by removing the flattening filter. The high dose rate provided by the FFF photon beams decreases the beam-on-time and improves clinical throughput [1–3]. These features can be effectively used for minimization of intrafraction motion of lung tumors [4].

Sublethal damage repair (SLDR) is induced over times ranging from several minutes to hours after irradiation of DNA by photon beams. The effect of cell death decreases with increased dose-delivery time [5, 6]. For the treatment of tumors, such as lung cancers that exhibit intrafraction motion, it takes approximately 10 min or longer to deliver the dose per fraction, depending on the irradiation technique of dose-delivery. Several techniques can be employed, such as stereotactic body radiation therapy (SBRT) [7, 8], real-time tumor tracking radiation therapy [9], and respiratory-gated radiation therapy [10]. Therefore, it is necessary to evaluate the effect of dose-delivery time on the radiation therapy techniques.

Hawkins proposed the microdosimetric kinetic model (MKM) in 1994 [11]. The MKM was developed by considering repair-misrepair [12], and lethal and potentially lethal [13] models. The model was developed to reveal the relationship between the surviving fraction (SF) and absorbed dose (D) by calculating the total energy deposition and analyzing the dose-rate effect [14]. Matsuya et al. reported MKMs which include various irradiation methods with photon beams [15, 16]. In addition, they evaluated DNA damage using the MKM and modified MKM; changes in the amount of DNA per nucleus during irradiation was analyzed [17]. The modified MKM provided a better SF estimate, which is used in the high dose range, compared with the previous MKM. Therefore, the dependence of the SF as a function of dose-delivery time was evaluated in the high dose range using the modified MKM.

Previous studies suggested that the RBE of the FF beams can be explained using the MKM [18, 19]. On the other hand, to the best of our knowledge, several studies investigated the dependence of RBE of FF beams with increased dose-delivery time. The dependence of the RBE of the FFF beams as a function of dose-delivery time has not been reported.

In this study, using the MKM, we investigated the effect of dose-delivery time for both the FF and FFF beams.

## Materials and methods

### MKM

The nucleus of the considered cell is divided into several hundred independent areas, which are called domains. Potentially lethal lesions (PLLs) emerge after irradiation of the domains in the MKM [12]. The PLLs are classified into four categories according to their transformations: (I) irreparable lethal lesion (LL) that emerges through a first-order process (*a* is the transformation rate constant); (II) converted to LLs through a second-order process (rate constant of the transformation; (III) repaired through first-order process (*c* is the transformation rate constant); (IV) lesions that resist becoming LLs for a period of time *t*_*r*_, after which they do become LLs and are not repairable. In the MKM, the PLLs are supposed to be DNA double-strand breaks. The average number of LLs per cell nucleus (*L*_n_) was defined as:

$$L_{n}=N\langle L\rangle =N\langle A\langle z\rangle +B\langle z^{2}\rangle \rangle$$

$$=(\alpha_{0}+\gamma \beta_{0})D+\beta_{0}D^{2}$$

$$=\left(\alpha_{0}+\frac{y_{D}}{\rho \pi r_{d}^{2}}\beta_{0}\right)\;D+\beta_{0}D^{2}$$

=−lnS (1)

$$\alpha_{0}=NA$$ (2)

$$\beta_{0}=NB$$ (3)

$$\gamma =\frac{y_{D}}{\rho \pi r_{d}^{2}}$$ (4)

where *z* is the specific energy deposited in the domain [Gy], *N* is the number of domains, *A* and *B* are coefficients, *r*_d_ is the radius of the domain (0.5 μm), *ρ* is the density of the domain (1.0 g/cm^3^), *D* is the absorbed dose [Gy], and *y*_D_ is the dose-mean lineal energy [keV/μm]. The parameters *α*_0_ and *β*_0_ were obtained by single instantaneous irradiation using a linear-quadratic (LQ) model. Matsuya et al. have modified the MKM while considering various irradiation schemes with photon beams. They considered changes in the amount of DNA per nucleus during irradiation (modified MKM). The equation that defines the modified MKM is defined as:

$$\begin{array}{l} \underset{N\to \infty }{lim}\left(-ln\;S\right)=\underset{N\to \infty }{lim}\sum_{n=1}^{N}\left[\left(\alpha_{0}+\gamma \beta_{0}\right)\dot{D}T+\beta_{0}{\left(\dot{D}\Delta T\right)}^{2}\right]\\ \;+2\;\underset{N\to \infty }{lim}\sum_{n=1}^{N-1}\sum_{m=n+1}^{N}\left\{\beta_{0}\left[e^{-\left(m-n\right)\left(a+c\right)\Delta T}\right]\right\}{\left(\dot{D\Delta T}\right)}^{2} \end{array}$$

$$=(\alpha_{0}+\gamma \beta_{0})\dot{D}T+\beta_{0}\left\{\frac{2}{{\left(a+c\right)}^{2}T^{2}}[(a+c)T+e^{-(a+c)T}-1]\right\}{\dot{D}}^{2}T^{2}$$ (5)

Therefore, we have

$$-ln\;S=(\alpha_{0}+{\gamma \beta }_{0})D+{F\beta }_{0}D^{2}$$

$$=\alpha D+\beta D^{2}$$ (6)

$$F=\frac{2}{{\left(a+c\right)}^{2}T^{2}}[(a+c)T+e^{-(a+c)T}-1]\;(T<t_{r})$$ (7)

$$D=\dot{D}T$$ (8)

$$\alpha =\alpha_{0}+\gamma \beta_{0}$$ (9)

$$\beta =F\beta_{0}$$ (10)

where $\dot{D}$ is the dose rate [Gy/min], *T* is the dose-delivery time [min], and *F* was obtained using the Lea–Catcheside time-factor *G* [20]. One can see that the value of *α* is affected by the dose-mean lineal energy *y*_D_ (Eq 7).

### Monte Carlo simulations performed using the particle and heavy ion transport code system (PHITS)

**Fig 1:**
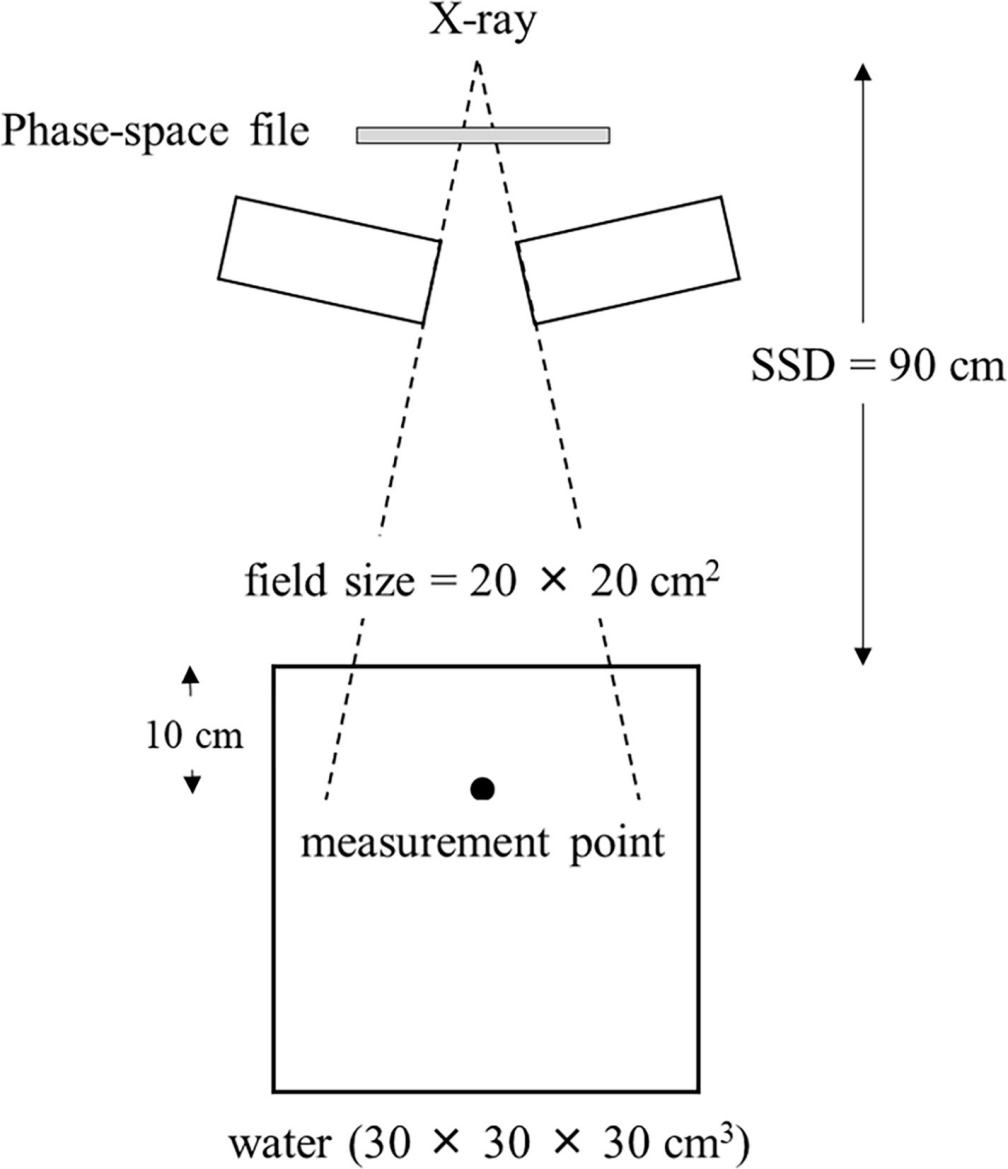
Irradiation geometry for the Monte Carlo calculations for both 6 MV FF and FFF beams.

The BEAMnrc code is based on the EGSnrc platform and is optimized for modeling the treatment head of radiotherapy linear accelerators [21]. This code includes several geometry and source subroutines, along with variance reduction techniques to enhance simulation efficiency [22]. PHITS can address photons, electrons, positrons, neutrons, and heavy ions [23]. In this study, International Atomic Energy Agency (IAEA) phase-space files for the Varian TrueBeam linear accelerator (Varian Medical Systems, Palo Alto, USA) were used to simulate the 6 MV FF and FFF beams. Fig 1 shows the irradiation geometry for the 6 MV FF and FFF beams, which is employed in the PHITS simulations. The measurement point was located at a 10 cm depth in the water-equivalent phantom material. The dose-mean lineal energy *y*_D_ [24–26] was calculated as:

$$y=\frac{\varepsilon }{l}$$ (11)

$$y_{D}=\frac{\int y^{2}f(y)dy}{\int yf(y)dy}=\frac{\int yd(y)dy}{\int d(y)dy}$$ (12)

where *ε*, *l*, *f*(*y*), and *d*(*y*) are the energy deposited in a domain, mean chord length, probability density of the lineal energy, and dose distribution of the lineal energy, respectively.

### Determination of biological parameters for the MKM by analyzing NCI-H460 lung cancer

Human non-small-cell lung cancer NCI-H460 cells were used to determine the MKM parameters. The parameters *α*_0_ and *β*_0_ were obtained using the LQ model [27], while the values of *α* and *β* were determined using Eqs (9) and (10), respectively. The PLL repair rate (*a* + *c*) was equated with the first-order rate constant *λ*, which was calculated using the DNA repair half-time *T*_1/2_. The value of the DNA repair half-time *T*_1/2_ for H460 was already reported [28, 29]. The characteristic damage repair rate λ [20, 30] was defined as:

$$\lambda =\frac{ln\;2}{T_{1/2}}$$ (13)

Although each of the DNA repairs occurred with a different rate constant, the DNA repair rate was simply calculated as λ in the MKM. Table 1 shows the calculated parameters for the NCI-H460 cells used in the MKM simulations.

**Table 1:** MKM simulation parameters obtained using NCI-H460 cells.

Parameters	Values
α_0_ (*Gy*^−1^) (FF)	0.24 ± 0.19
α_0_ (*Gy*^−1^) (FFF)	0.21 ± 0.11
β_0_ (*Gy*^−1^) (FF)	0.06 ± 0.03
β_0_ (*Gy*^−1^) (FFF)	0.07 ± 0.02
a + c (*h*^−1^)	0.46
*γ*_*FF*_	2.96
*γ*_*FFF*_	2.98
*ρ* (*g*/*cm*^3^)	1.00
*r*_*d*_ (μ*m*)	0.50

### SF and RBE calculations for both FF and FFF beams using the MKM

The absorbed dose on the NCI-H460 cells was varied from 0 Gy to 8 Gy by applying single instantaneous irradiation, and the dose-delivery time was varied from 0 to 60 min. Moreover, the RBEs for both the FF and FFF beams were calculated in order to evaluate the effect of the dose-delivery time. For the FF and FFF beams, the RBE was defined using instantaneous irradiation (*T* = 0) as a reference [31] (Eq 14):

$$\begin{array}{c} RBE=\left[\frac{D_{T=0}}{D_{T}}\right]\\ ={\left(\frac{\sqrt{\alpha_{T=0}^{2}+4\beta_{T=0}S_{T=0}}-\alpha_{T=0}}{2\beta_{T=0}}\right)}^{-1}\cdot \left(\frac{\sqrt{\alpha^{2}+4\beta S}-\alpha }{2\beta }\right) \end{array}$$ (14)

## Results

### Microdosimetric energy distribution calculations y_D_

**Fig 2:**
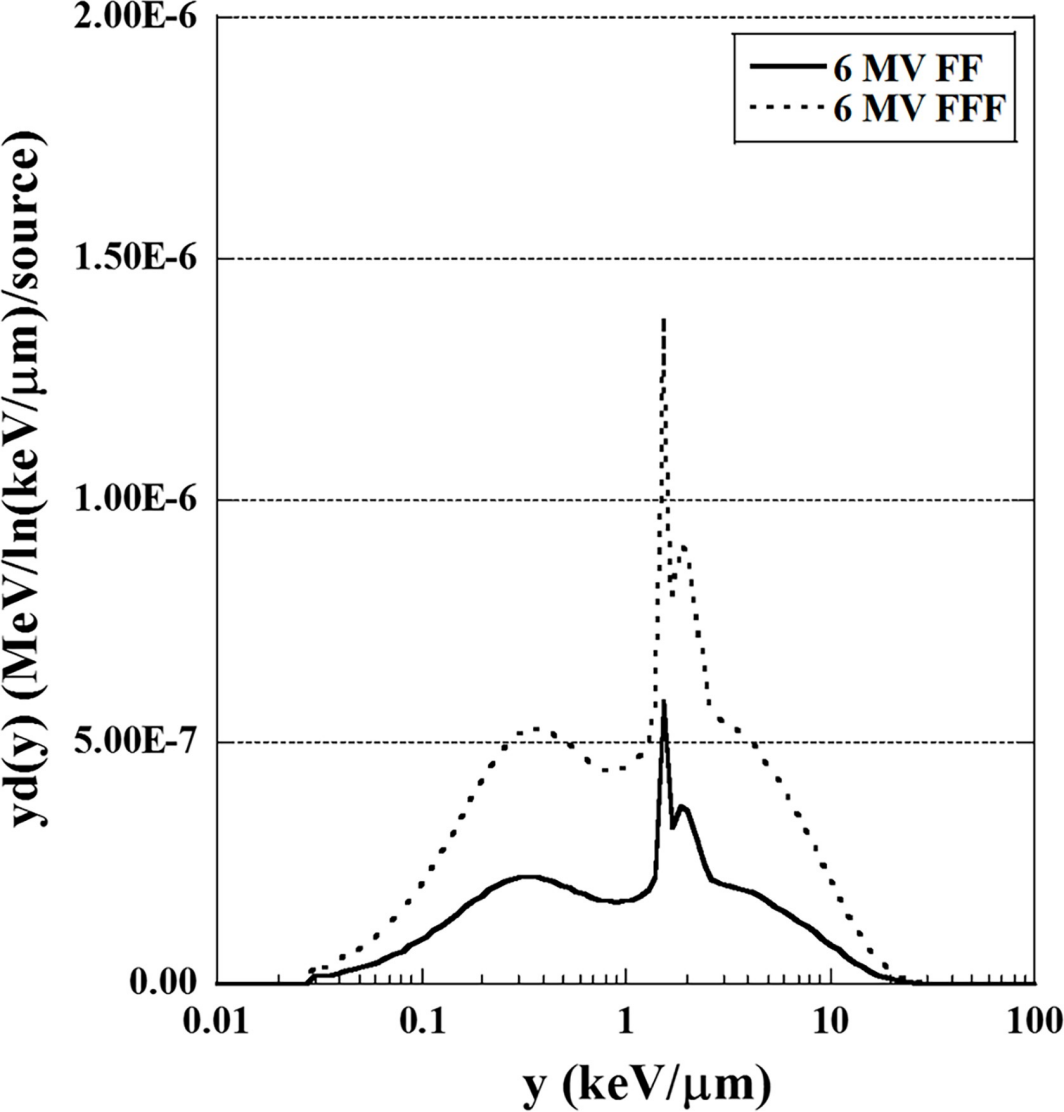
Microdosimetric energy distributions *y*_*D*_ as a function of *y* for 6 MV FF and FFF beams at a depth of 10 cm in a water-equivalent phantom material.

The energy distributions of the FF and FFF photon beams in a domain were calculated using PHITS. The *y*_*D*_(*y*) distributions for the FF and FFF photon beams are shown in Fig 2. One can notice that the *y*_*D*_(*y*) distributions of the FF and FFF beams show similar behavior. The *y*_*D*_(*y*) distributions were used to calculate the dose-mean lineal energy *y*_D_ using Eq (12).

**Fig 3:**
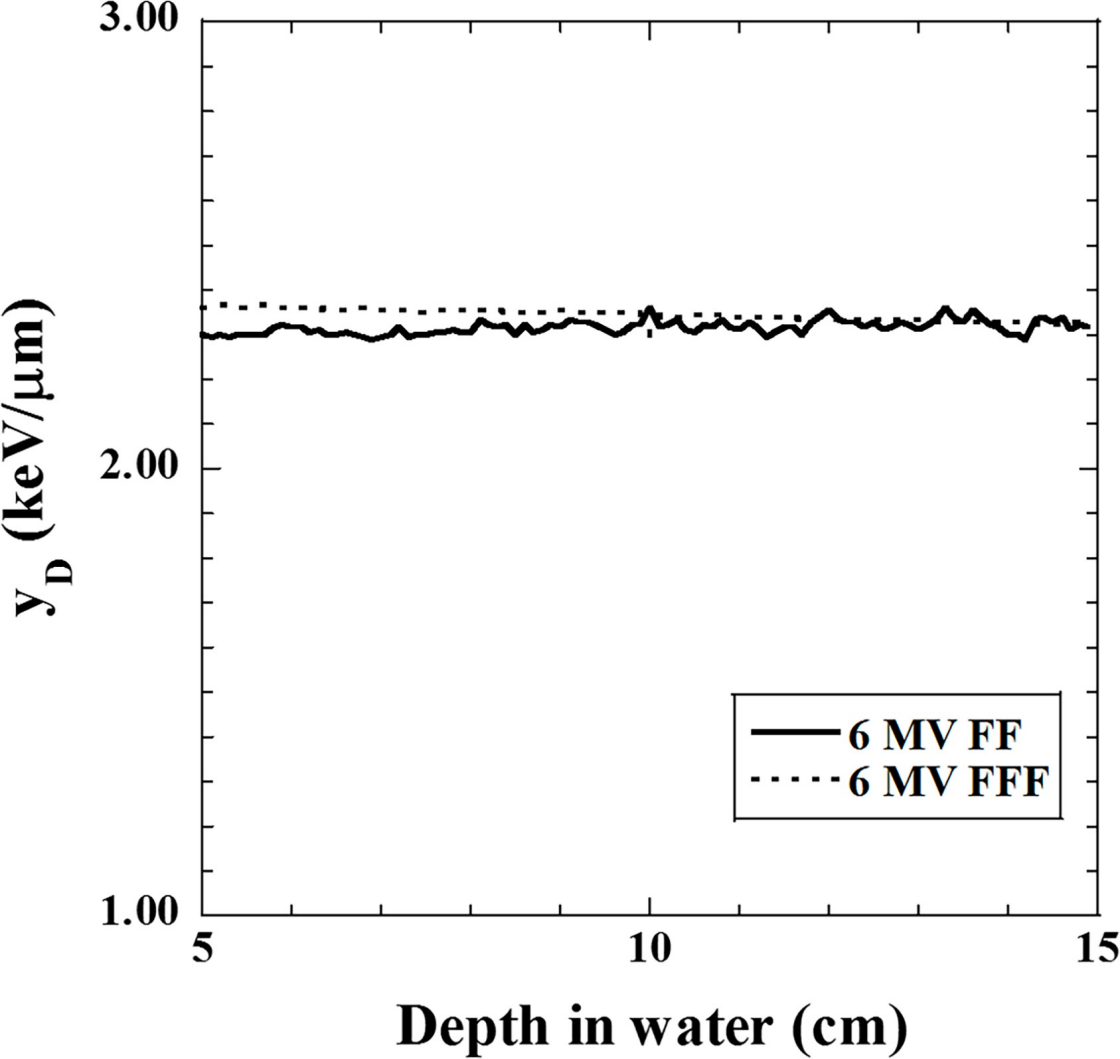
Relationships between the depth in water and dose-mean lineal energy *y*_D_ for the 6 MV FF and FFF beams.

Fig 3 shows the relationships between the measured depth in water and dose-mean lineal energy *y*_D_ for both FF and FFF beams. One can see that the value of *y*_D_ was affected by the depth. Therefore, *y*_D_ was averaged over depths ranging from 10 cm to 13 cm. Table 2 shows the average *y*_D_ value for the FF and FFF beams. The average *y*_D_ value was used for to calculate the SF and RBE using the MKM.

**Table 2:** Average value of the dose-mean lineal energy *y*_D_ for FF and FFF beams.

Dose-mean lineal energy	Value (mean ± standard deviation)
${y_{D}}_{FF}$	2.32 ± 0.01
${y_{D}}_{FFF}$	2.34 ± 0.01

#### Effect of the dose-delivery time on the SF for the FF and FFF beams using the MKM

**Fig 4:**
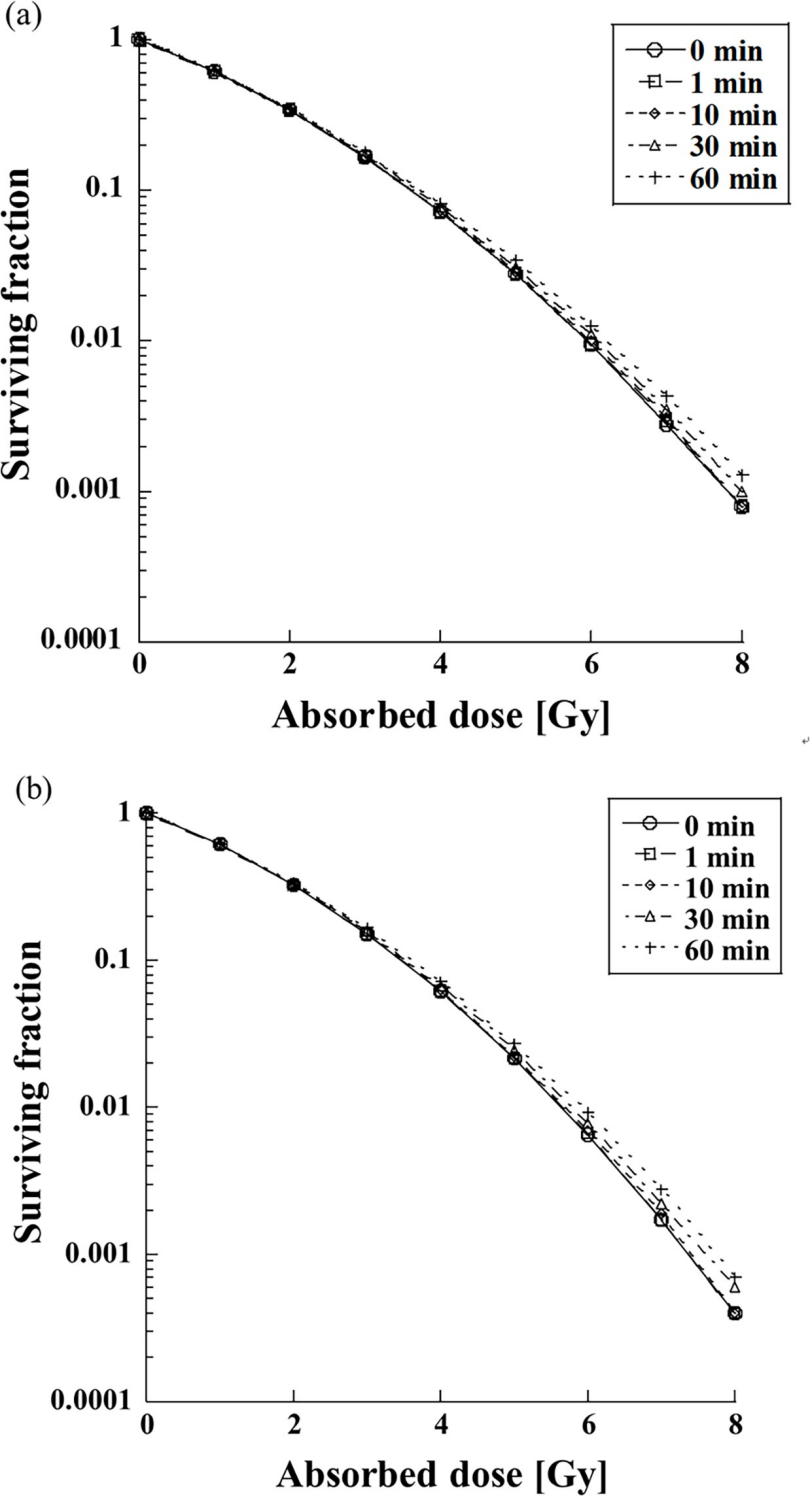
Effect of the dose-delivery time on the SF for FF (upper plots) and FFF (lower plots) beams for various dose-delivery times.

Fig 4 shows the dependence of SFs for the FF and FFF beams. Although there was no difference between the FF and FFF beams, the SF decreased with increased dose-delivery time. The difference between SFs for the FF and FFF beams was emphasized when the dose-delivery time was 30 min or longer.

**Fig 5:**
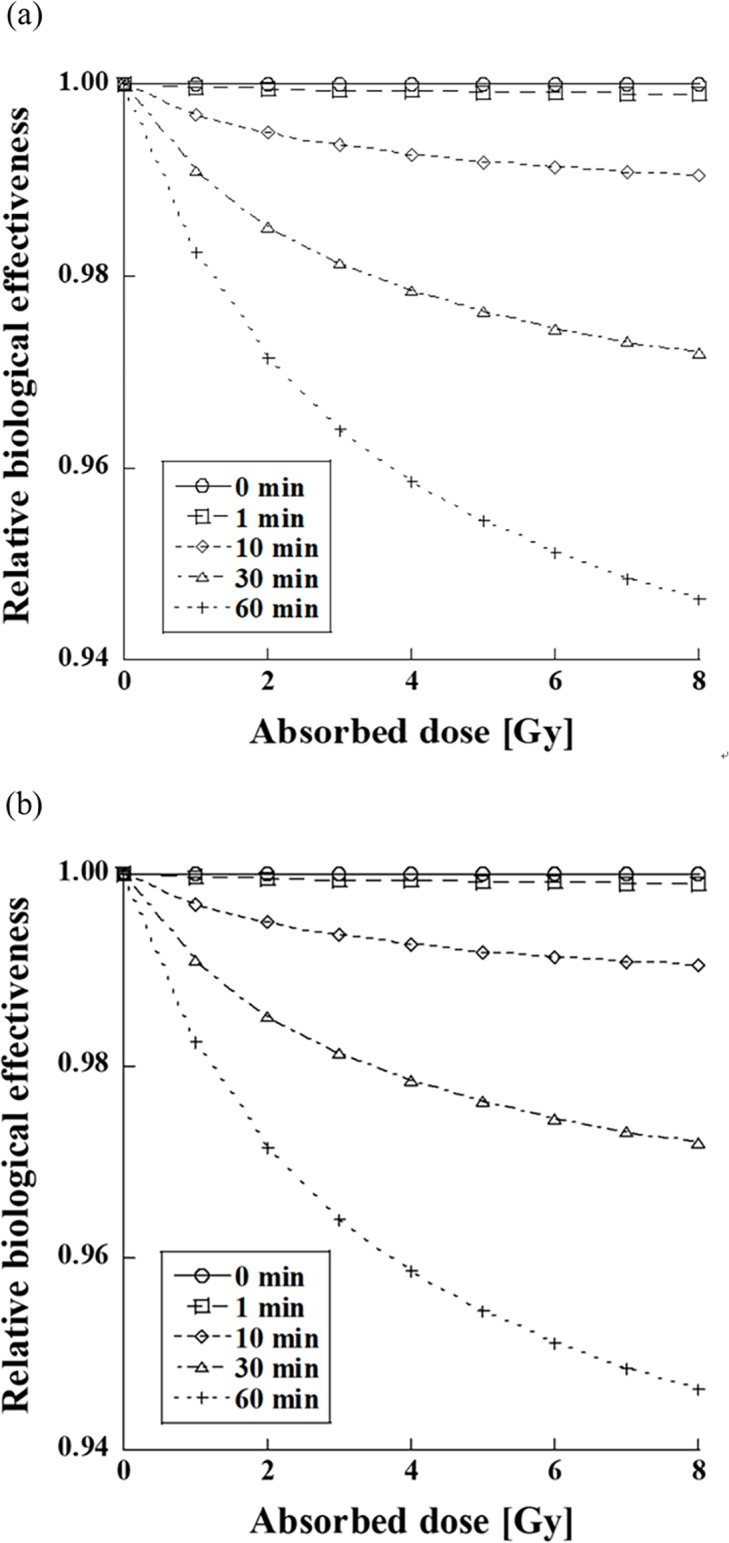
Effect of the dose-delivery time on the RBEs of the FF (upper plots) and FFF (lower plots) beams.

Fig 5 shows the RBE of the FF and FFF beams for different dose-delivery times. The RBE of the FF beam decreased to 0.998 and 0.974 after 5 min and 60 min, for a dose of 2.0 Gy, respectively. For a dose of 8.0 Gy, it decreased to 0.996 and 0.951 after 5 min and 60 min, respectively. The RBE of the FFF beam decreased to 0.997 and 0.972 after 5 min and 60 min for a dose of 2.0 Gy, respectively. For a dose of 8.0 Gy, it decreased to 0.995 and 0.949 after 5 and 60 min, respectively. The RBEs of the FF and FFF beams decreased by ~5% when the dose-delivery time was 60 min.

## Discussion

The RBEs of the FF and FFF beams for various dose-delivery time values obtained from the SF using the modified MKM were calculated in this study. No significant differences between the RBEs of FF and FFF beams were found, and both RBEs decreased with increased dose-delivery time. Both RBEs were affected when the dose-delivery time was 30 min or larger, where they decreased by more than 2% as a function of the absorbed dose. The dose-delivery time for photon irradiation in clinical applications depends on the irradiation techniques, which include SBRT, tumor tracking, and respiratory-gated radiation therapy. If the dose-delivery time is prolonged in order to apply a large dose of about 8 Gy, it is possible that the treatment effects with photon irradiation are reduced due to SLDR. Therefore, the dose-delivery time should be considered as an additional factor for achieving successful photon treatment. The FFF beams could provide better clinical throughput since the FFF beams have high dose rate compared to the FF beams.

There was no significant diferrence in the effect of dose-delivery time from FF and FFF since the dose-mean lineal energy *y*_D_ values of the FF and FFF were nearly equivalent in this study. However, the comparison between the FF and FFF beams shows that FFF beams provide better clinical throughput. In other words, the FFF beams could decrease the required dose-delivery time [31, 32]. Therefore, the FFF beams could be employed for effective irradiation in radiation therapy due to their smaller dose-delivery time.

**Fig 6:**
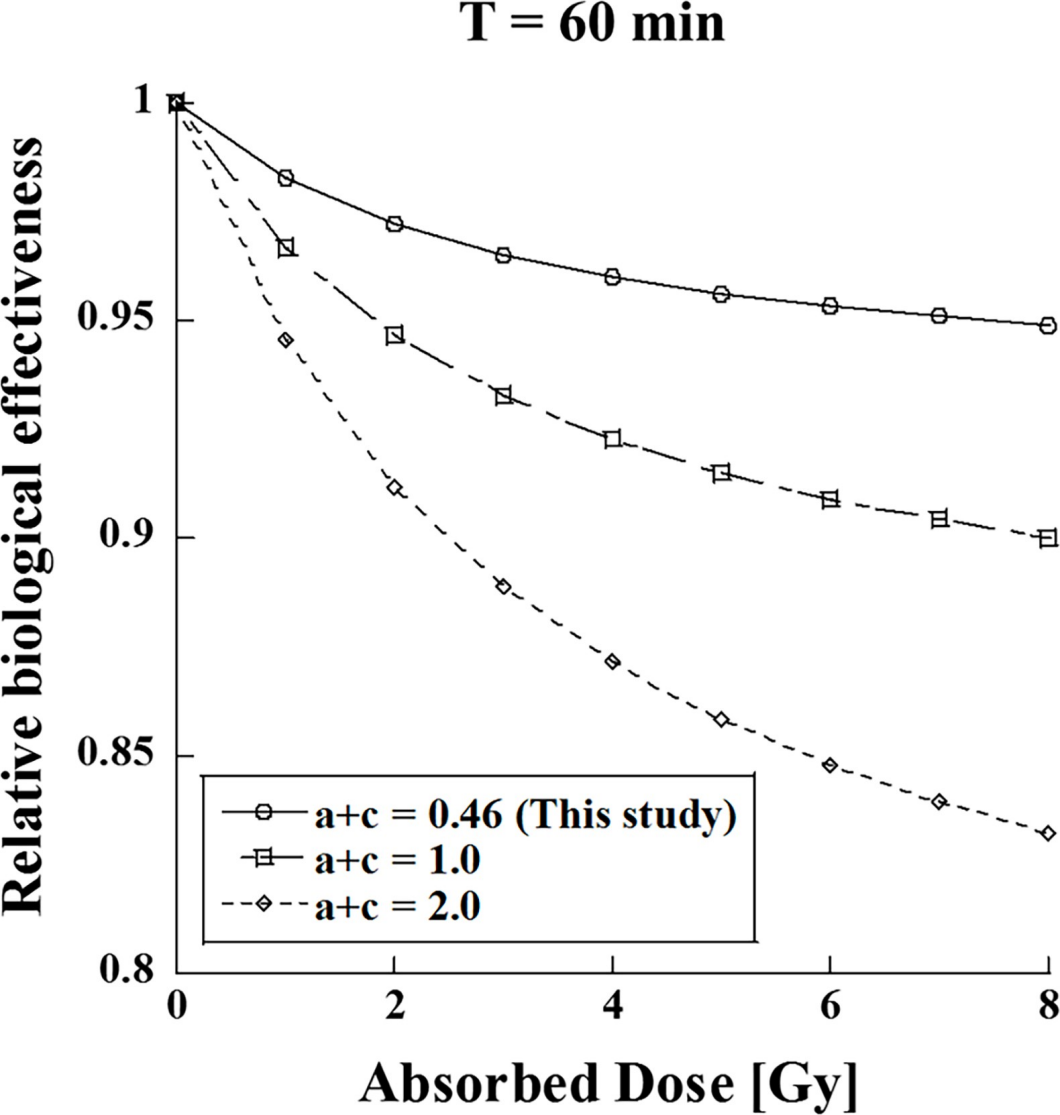
Effect of relative biological effectiveness as a function of the cell-specific repair rate (*a* + *c*) (*T* = 60 min).

Fig 6 shows the dependence of the RBEs for various cell specific values (*a* + *c*) (*T* = 60 min). One can see that the RBE is affected by the value of (*a* + *c*). The effect of the dose-delivery time was significantly different by more than 10% (Fig 6). As the cell specific value (*a* + *c*) depends on the type of tumor cell [33, 34], it is necessary to evaluate how the dose-delivery time affects each type of tumor cell.

However, only in-vitro studies on NCI-H460 cells were performed in this study in order to evaluate the effect of the dose-delivery time. It is necessary to examine the effect of the dose-delivery time by performing in-vivo studies on various tumor cells. In addition, the effect of the dose-delivery time for clinical irradiation applications, including multiple-field therapy, intensity-modulated radiation therapy, and volumetric-modulated arc therapy should be investigated.

## Conclusions

In this study, it was shown that the dose-delivery time affects the RBE during photon irradiation. The effects of the dose-delivery time are important and should be considered in radiation therapy.
